# Incremental Benefits of Multiple Tobacco Control Interventions: A Factorial Randomized Control Trial

**DOI:** 10.31557/APJCP.2020.21.7.1905

**Published:** 2020-07

**Authors:** Divya Persai, Anup Karan, Rajmohan Panda

**Affiliations:** *Indian Institute of Public Health, Public Health Foundation of India, Delhi NCR, India. *

**Keywords:** Community intervention, health system intervention, primary care, randomized control trial, tobacco control

## Abstract

Tobacco control requires a comprehensive approach. The present study aims to examine the incremental effectiveness of health systems intervention when combined with other interventions in enhancing knowledge and practices of physicians in tobacco cessation.

**Methods:**

A randomized control trial was conducted among 437 physicians in 12 districts of two states of India in 2011-13. The interventions consisted of Health Systems (H), Community (C) and Youth intervention (Y). Administrative Blocks /Mandals were randomly assigned to one of the three interventions (HC /HCY/HY) and control units. The health system intervention consisted of training physicians and developing a system of patient support and supervision for tobacco cessation. The primary outcome was change in knowledge and practices of physicians in tobacco cessation. Logistic regression model was applied to assess the impact of single and combination of interventions.

**Results:**

An increase in knowledge was observed on effects of tobacco on adverse birth outcomes, advice on NRT and, information provided on chronic disease management among physicians in HC, HY and HCY intervention units compared to control units from pre-intervention to post-intervention. Statistically significant change was observed in knowledge of physicians on effects of tobacco on adverse birth outcomes in HC (OR- 4.75, p-0.02) and HCY (OR- 5.08, p-0.04) intervention units.

**Conclusions:**

HCY intervention was most effective in enhancing knowledge and practices of physicians in tobacco cessation. Our study suggests that individual tobacco control interventions when combined together has an incremental effect and increases the likelihood of provision of tobacco cessation services in primary care.

## Introduction

Tobacco use is a leading cause of preventable premature death in the world today, claiming 100 million premature lives per year (WHO, 2008). Tobacco use is determined by multiple factors and attempts to control the epidemic requires a comprehensive approach one that optimizes synergy from applying a mix of educational, clinical, and social strategies (CDC, 2014).The Framework Convention on Tobacco Control has catalyzed tobacco control efforts to reduce both supply and demand for tobacco (WHO, 2003). Most strategies to reduce tobacco demand fall into two broad categories i.e. Clinical and population-level interventions. Research has shown greater effectiveness with multi-component interventional efforts that integrate the implementation of programmatic and policy initiatives in tobacco control to influence social norms and systems (National Cancer Institute, 2005; National Cancer Institute 1995). Health systems intervention plays an important role in tobacco control. There is sufficient evidence that a brief advice from physicians is effective in educating patients about harmful health effects of tobacco and promoting cessation (Rubak et al., 2005; Fiore et al., 2008). Individual-level health care provider interventions involve one-to-one interactions between patient and a provider often within a clinical environment. However, clinical services can also extend to proximal large systems (e.g., the community), and are well suited for addressing the health needs of the individual and the community (Ockene et.al., 2008; Zhu et al., 2012).

Community-wide interventions attempt to change tobacco use in population and have increasingly begun to focus on influencing policies that promote tobacco cessation (Zhu et al., 2010; Cummings et al., 1999). Health system and community interventions have been reported as having identifiable, unique effects on tobacco cessation. These interventions also have incremental effect when applied together in the real-world settings (Zhu et al., 2010). However, quantitative knowledge base on the impact of multiple tobacco control interventions is methodologically weak as well as limited in scope in developing countries. To our knowledge, none of the studies employed a true randomized design to evaluate a package of interventions in tobacco control in health care settings in India. To fill the gap, a factorial randomized control trial was designed to evaluate whether health system intervention and health system intervention when combined with different tobacco control interventions are more effective in changing the knowledge and practices of physicians in tobacco cessation as compared to stand-alone intervention. This study evaluates the effect of a package of interventions conducted at the health system, community and school level and highlights ‘what works’ to increase knowledge and practices of physicians in tobacco cessation. 

## Materials and Methods

*Study Design*

The study was a randomized controlled trial with a factorial design. The factorial design enabled us to conduct an efficient evaluation of the impact of each intervention and simultaneous investigation of multiple interventions. The design of the current study is shown in Figure 1.

The interventions were made at various levels i.e. Health Systems, community and school going youths (10-16yrs). Single and multiple interventions were planned to capture the single and incremental effect of health system intervention, 3 combined interventions i.e. Health System and Community (HC), Health System, Community and Youth (HCY), and Health System and Youth (HY) and control (where no interventions were planned).

*Study Settings*

Two surveys were conducted in 12 districts of Andhra Pradesh and Gujarat in India in August 2011 (pre-intervention) and August 2013 (post-intervention). These states are high tobacco burden states and have distinct geographical distribution. The surveys were administered among physicians working in health facilities providing primary care. Health facilities providing primary care in India include Primary Health Centres (PHC) and Community Health Centres (CHC), which serve as the first point of contact for patients with a healthcare professional in the public sector.

*Randomization, sample size, and power analysis*

A total of six districts in each state were selected excluding the districts covered by the National Tobacco Control Programme (NTCP) of India. The districts represent different geographical regions of the states. Mandals/Talukas were the Primary Sampling Units (PSUs) randomly assigned to one of the four interventions and control conditions. The proportion of revenue districts /blocks selected for each single and combined intervention and control was 3:4 respectively. 

 A total of 140 Blocks and Mandals in 12 districts of two states were randomly assigned to interventions and control conditions by the authors. In each district, 30 health facilities were selected through systematic random sampling. Physicians were selected through simple random sampling. The estimated change expected in the practices of physicians from pre-intervention to post-intervention was 12%. The desired sample size was calculated with reference to the primary outcome, an attrition of 10% and 80% power, with an alpha error of 5% (double tailed). Pre-randomization matching of PSUs was considered in order to improve balance on important observable characteristics between intervention and control units. 

*Data Collection*

The data for this study were collected through two surveys conducted among 191 physicians (Intervention: 106(55%); Control: 85(45%) in base-line and 236 physicians (Intervention: 116 (49%); Control: 120(51%)) post-intervention. Although the numbers of physicians were different in the pre-intervention and post-intervention because of transfers of the officers, we assumed that the institutional memory of the intervention made at the facility level has been transferred to the new officers joining the facilities. Consenting physicians completed a semi-structured questionnaire. The questionnaire was administered by trained interviewers hired from a survey agency. The questionnaire comprised of four sections – a) Background characteristics, b) Practices of physicians in tobacco cessation c) Knowledge of physicians in health effects of tobacco, d) Attitude towards tobacco cessation. 

*Interventions*

Interventions in the present study consisted of three levels, administered independently in a few PSUs and simultaneously in other PSUs. Health system intervention aimed to improve access to effective tobacco prevention and cessation services (behavioral and pharmacological) for patients and tobacco users. The health system intervention consisted of training physicians in tobacco cessation, use of educational material for counselling patients and developing a system of patient support and supervision for tobacco cessation. Community intervention aimed to create an enabling environment for tobacco control through advocacy activities. It provided a set of strategies for publicizing the tobacco problem to influence community members to support efforts to prevent tobacco use. Activities included conducting community outreach activities to motivate communities to adopt smoke-free norms, providing communication support materials for community mobilizing meetings, and technical support to local non-government and community-based organizations. Youth intervention aimed at preventing tobacco use among school-going youths (10-16 years) through health education, skill building, empowerment and implementation of smoke-free policies. Table 1 illustrates the description of each intervention.

*Control Units*

Mandals/ Talukas in the control units did not receive any intervention. Pre-intervention and post-intervention studies were conducted to assess the change in knowledge and practices of physicians in tobacco cessation. Physicians’ demographics such as age, education, place of work and years of experience were also assessed in both the surveys in control units.

*Outcome Measures*

The primary outcome was defined as knowledge on health effects of tobacco and information provided on effects of tobacco on health conditions by the physicians. These measures included: Knowledge of physicians on health effects of tobacco on 1) heart diseases, 2) adverse birth outcomes and 3) advice on Nicotine replacement therapy (NRT). Practices in tobacco cessation were gauged by information provided by physicians on chronic disease management and during antenatal care. All these outcomes were measured in ‘Yes’, ‘No’ format during the pre-intervention and post-intervention studies. 

*Control variables*

Knowledge of physicians and information provided by them to the patients may also confound with various individual and demographic characteristics of physicians. In the impact analysis, socio-demographic factors such as age, education, place of work and years of experience of physicians were controlled. Work experience of physicians was calculated by subtracting 22 yrs (12 years of higher secondary education, 5years of medical education and additional 5 years) from age. Physicians in the interventions and control PSUs did not differ statistically in background characteristics during the base-line survey (Table 2). More than two-third of the physicians in the intervention and control units were males below 35 years. All the physicians had a medical degree and more than half of them have an average work experience of 17 years.

*Data Analyses*

Considering the two periods, pre-intervention and post-intervention and two population groups (treatment and control groups), the effects of the intervention can be estimated by specifying the following regression equation:

$y_{\mathrm{it}}=\alpha +\beta_{1}d_{t}+\beta_{2}d_{t}+\beta_{3}d_{t}\mathrm{.T}+{\mathrm{\varepsilon i}}_{t}$

(1)

Where: *y*_jt_ is the outcome of interest for individual physician *i* in time period t and *d*_T_ is a dummy for physicians who received treatment. d_t_ is a dummy variable for the post-intervention time period (t_1_) as against the pre-intervention time period (t_0_). The effect of the intervention is given by which is the coefficient of the interaction between the treatment and the post-treatment period i.e. dummy. The dummy equals unity for the treatment group in the post-intervention period and captures the effect of the treatment over time. *ɛ*_i_ is the error term. Since equation (1) does not control for any socio-economic characteristic (confounders), *β*_3_ represents a ‘*simple difference*’ (or unadjusted for covariates) in the mean outcome of the treatment group. 

We assessed the impact of intervention after controlling for social and demographic characteristics of physicians. We also used district level fixed effects to control for any district- level fixed unobserved factors. Finally, since our outcome indicators are categorical, instead of using a ‘linear probability model’ we used ’limited dependent variable model’ as mentioned in equation (2).

$y_{\mathrm{it}}=\alpha +\beta_{1}d_{t}+\beta_{2}d_{T}+\beta_{3}d_{t}\mathrm{.T}+{\mathrm{\varepsilon i}}_{t}$

 (2)

Where y_ijt_ and X_ijt_ respectively represents outcome and a vector of social and demographic characteristics of individual physician i, living in district j in time period t_1_. The term *ǌ *stands for district -level fixed effects. Inference with respect to the impact of the intervention is based on point estimates of the odds ratio on the outcome relative to knowledge and practices of physicians with 95% confidence intervals and corresponding standard test results.

## Results

*Simple Difference*

Supplementary Table 1 illustrates a simple difference in outcome indicators between pre and post-intervention across different interventions and control units. For most of the outcome indicators, any type of intervention reflects a positive change in knowledge and practice of physicians in tobacco cessation in intervention units as compared to the control units. An increase in knowledge on effects of tobacco on adverse birth outcomes (32%), advice of NRT (13%) and information provided on chronic disease management (8%) was observed among physicians in health system and community (HC) intervention units as compared to control units (Adverse birth outcome: 8%; Advice on NRT:-8%; Information on chronic disease management: 2%). 

An increase in the knowledge of physicians on the effects of tobacco on heart disease across various interventions and control groups was observed. The increase in the knowledge was larger for health system (28%) and health system combined with youth intervention units (HY) (29%) compared to control units (20%). However, when health system intervention was combined with community and youth intervention (HCY) the change in knowledge of physicians about effects of tobacco on heart diseases is lower in intervention as compared to the control. The simple difference results also reflect a couple of negative changes and lower positive changes in the intervention groups compared to those in the control units. However, these changes reflect only unadjusted (for social and demographic characteristics of physicians) results. 

*Impact of intervention*

The impact of the intervention on all the outcome measures was measured using equation 2. Table 3 illustrates results on impact of combined intervention. This is followed by results on different types of intervention separately reported in Table 4. For better readability, we only present estimates of odds ratio (OR) of the interaction term (*d*_t_*.d*_T_) in equation (2) which represents the impact of the intervention. 

*Impact of combined intervention*

Almost all the outcomes, except knowledge of physicians on effects of tobacco on heart diseases (OR-1.01), reflect positive changes after the combined intervention (Table 3). The highest change was observed in the outcome ‘Knowledge of physicians on effects of tobacco on adverse birth outcomes’, which reflected 158% increase (OR 2.58) due to the combined interventions. Knowledge of physicians on advice on NRT too increased by 91%, although this was not statistically significant at 95% significance level. Both the outcomes related to the practice of physicians also changed positively (information provided during antenatal care by 39% and chronic disease management by 25%) but did not stand statistical significance. 

*Impact of different interventions*

*1. Health system and Community (HC):* An increase in knowledge of physicians on advice on NRT (85% increase) and information provided by physicians to patients on chronic disease management (207%) was observed. The change in knowledge of physicians on effects of tobacco on adverse birth outcomes was significant (OR-4.75; p-0.02).

*2. Health system and Youth (HY): *A positive change in knowledge of physicians on advice on NRT (OR-2.53) and effects of tobacco use on heart diseases (OR-1.91) was observed. An increase in information provided by physicians on chronic disease management (87%) was observed. However, the results were not statistically significant. 

*3. Health system + Community+ Youth (HCY):* An increase in knowledge and practices of physicians was observed for all the outcomes except information provided by physicians on effects of tobacco on chronic disease management (OR-0.79). The change in knowledge of physicians on effects of tobacco on adverse birth outcomes was statistically significant (OR-5.08; p-0.04).

**Table 1:** Health System, Community and Youth Intervention

Health System Intervention	Capacity building of physicians in tobacco cessation Development of culturally appropriate education materials Development of sustainable system in tobacco control by establishing tobacco cessation and a network of trained and skilled experts at the district level known as district resource hubs
Community Intervention	Capacity building of Non-Government Organization and Community Based Organizations in tobacco control Development of education, information and communication material in tobacco control Conduct of community meetings to generate support and awareness for tobacco control initiatives Community outreach activities were conducted to motivate communities to adopt smoke-free norms
Youth Intervention	Conducting sensitization meetings and workshops with school administration and youths in the schools Facilitation of implementation of tobacco free school policies by student peer leaders and teachers

**Table 2:** Background Characteristics of Physicians

		Pre-intervention % & CI	Post-intervention % & CI
Gender			
Male	Intervention	71 (CI:0.62-0.81)	72 (CI:0.63-0.79)
	Control	70 (CI:0.63-0.78)	64 (CI:0.57-0.72)
		P value-0.89	P value- 0.23
Female	Intervention	29 (CI:0.22-0.37)	28 (CI:0.18-0.38)
	Control	28 (CI:0.18-0.38)	35 (CI:0.27-0.43)
		p-0.86	p-0.21
Location			
Urban	Intervention	12 (CI:0.06-0.17)	64 (CI:0.55-0.72)
	Control	10 (CI:0.04-0.17)	48 (CI:0.40-0.56)
		p-0.79	p-0.01
Rural	Intervention	89 (CI:0.82-0.93)	34 (CI:0.25-0.42)
	Control	88 (0.82-0.96)	47 (0.39-0.55)
		p-0.78	p-0.03
Years of Experience			
Less than 13 yrs	Intervention	61 (0.51-0.71)	59 (0.51-0.68)
	Control	61 (0.53-0.69)	54 (0.45-0.61)
		p-0.98	p-0.33
More than 13 yrs	Intervention	38 (0.27-0.48)	45 (0.37-0.53)
Mean Yrs of experience:13 yrs	Control	38 (0.29-0.46)	39 (0.30-0.47)
		p-0.98	p-0.30
Age			
Less than 35yrs	Intervention	62 (0.51-0.72)	58 (0.47-0.63)
	Control	61 (0.52-0.69)	61 (0.52-0.69)
		p-0.85	p-0.31
More than 35 yrs	Intervention	38 (0.27-0.48)	45 (0.37-0.53)
	Control	39 (0.31-0.44)	39 (0.30-0.47)
Mean age:35.1 yrs		p-0.85	p-0.31
*P*-value <0.05			

**Table 3:** Impact of Combined Intervention on Knowledge and Practices of Physicians in Tobacco Cessation

Outcome Variable	Adjusted OR
Knowledge on effects of tobacco on Heart Diseases	1.01 (CI:-0.46-2.25)
Knowledge on effects of tobacco on Adverse Birth Outcomes	2.58* (CI:-0.92-7.23)
Knowledge on advice on NRT	1.91 (CI: -0.76-4.77)
Information provided on chronic disease management	1.25(CI: -0.45-3.53 )
Information provided on effects of tobacco during antenatal care	1.39 (CI: -0.39-4.84)

Analysis was adjusted for age, gender, years of experience of MOs, place of work of MOs, and districts level effects. *P-value close to 0.05

**Table 4:** Impact of Different Interventions on Knowledge and Practices of Physicians in Tobacco Cessation

Health System+ Community (HC)				Health System + Youth (HY)			Health system + Community+ Youth (HCY)		
Outcome Variable	Adjusted OR	Standard Error	P value	Adjusted OR	Standard Error	P value	Adjusted OR	Standard Error	P-value
Knowledge on effects of tobacco on Heart Diseases	0.64	0.55	0.43	1.91	0.69	0.35	1.04	0.58	0.94
Knowledge on effects of tobacco on Adverse Birth Outcomes	4.75*	0.69	0.02	0.52	0.85	0.44	5.08*	0.8	0.04
Knowledge on advice on NRT	1.82	0.62	0.33	2.53	0.82	0.26	1.37	0.66	0.63
Information provided on effects of tobacco during antenatal care	0.82	0.81	0.8	0.59	1.01	0.61	3.54	1	0.21
Information provided on Chronic Disease management	3.07	0.79	0.43	1.59	0.87	0.59	0.79	0.75	0.76

**, *P*-value <0.05; Analysis was adjusted for age, gender, years of experience of MOs, place of work of MOs, and districts level effects.

## Discussion

Tobacco use is a broad population-based public health problem and warrants a population-based model for tobacco control with wider application of clinic-based intervention. Our work is distinguishable in that we evaluated the relative and incremental impact of multiple types of interventions in the context of change in knowledge and practices of physicians in tobacco cessation. Our study shows that a multi-component intervention involving health system and community intervention was most effective in enhancing physicians’ knowledge about health effects of tobacco and advice on NRT. This is particularly important as the lack of knowledge is a significant barrier which interferes with clinician assessment and treatment of tobacco users. 

Evidence suggest that most physicians rarely advise and assist smokers in quitting tobacco use due to a lack of training, skills, and confidence in tobacco cessation. (Muramoto et al., 2010; Desalu et al., 2000; Sonmez et al., 2015) An especially important finding in our study is that there was a significant increase in tobacco cessation practices of physicians from pre-intervention to post-intervention. Consistent with other studies, our study suggests that increasing self-efficacy of physicians through training and facilitating the practice of tobacco treatment skills will help foster implementation of tobacco cessation interventions by service providers at the local level (NCI, 2005; Panda et al., 2013; Ulbrict et al. 2006). 

Tobacco use during pregnancy is associated with increased risk of maternal and infant adverse outcomes (WHO, 2003). A study suggest that training in tobacco cessation appeared to be the most important predictors of tobacco intervention use in antenatal clinics (Althabe et al., 2013). Similarly, our findings indicate that physicians in the intervention units who were trained in tobacco cessation were more likely to inform patients about adverse birth outcomes during antenatal care as compared to control units. These results highlight the critical importance of an integrated effort among physicians for a continuum of care during antenatal care to encourage quitting tobacco use.

Low-cost community-based tobacco control models are effective in enhancing tobacco abstinence and cessation. Community perception of and buy-in to the value of tobacco control interventions is a critical step in the adoption of interventions and recommendations. It is widely accepted that decisions to consume tobacco are made within a broad social context where lack of knowledge, awareness, perception, and community norms play a critical role (Walker et.al, 2005).Our results are consistent with the findings of the study (Slama et al., 2005) which suggest that strengthening community action is important in the context of tobacco control as it facilitates the provision of cessation advice and treatment. Our findings indicate that higher increase in knowledge and practices in tobacco cessation was observed among physicians practicing in health facilities where combined health, community and youth intervention was delivered. Maintenance of tobacco cessation practices requires an increase in community capacity (Muramoto et al, 2000). The community intervention leads to increase in public awareness about the health risks of tobacco and thus enhance demand for tobacco cessation services whereas health system intervention leads to an increase in the provision of services in tobacco cessation by the physicians. Thus, intervention model which included community advocacy, youth anti-tobacco activities, and intervention by physicians, appears to be of value for increasing tobacco cessation practices in primary care.

Our findings also suggest that physicians practicing in intervention areas where only health system and youth interventions were undertaken were less likely to provide information on harmful health effects of tobacco. Research suggests that interventions that specifically target only adolescents and youths are unlikely to have the desired effect, especially if done in isolation. Literature suggests that school-based programs are more effective when combined with the health system and community-based efforts (Bigan et al., 2000; Worden et al., 1996). Our study also suggests that health system intervention when combined with youth and community intervention does lead to significant increase in knowledge of physicians in tobacco cessation.

A possible explanation behind the more modest magnitude and precision of effects of different interventions in our study studies rests on characteristics of the comparison group. Information, education, and communication activities were undertaken at the control units under the National Tobacco Control Program in the intervention districts which probably led to an increase in knowledge and practices of physicians in control areas to some extent.

The study has key strengths. Firstly, the RCT design was used to good effect in this study. The randomized design can be considered the best protection against confounding and selection bias (Rotman, 2002). By choosing a randomized controlled design, the intention of producing comparable groups of physicians who differ only in terms of their exposure to intervention during the study was assured, and several potential confounders or biases, such as geographical location, physicians’ professional experience could be removed. Secondly, by measuring effects of different interventions, it goes beyond many studies carried out in the past which assessed the benefits of individual training interventions on improving the knowledge and practices of physicians in tobacco cessation. Thirdly, the intervention used evidence-based practices to build the knowledge and practices of physicians in tobacco cessation in primary health care settings. The study is limited by the fact that attribution of individual intervention i.e. health system, community and youth intervention was not assessed. In addition, self-reported data was used and physicians willing to participate in the study could be more interested and more engaged in tobacco cessation activities. The study was carried out in limited primary health centers in specific intervention states in India thus findings cannot be generalized to entire country which may have similar health systems but may not have the same eco-system. 

Our results have important implications for research and future design of tobacco control interventions and program. The results of this study can inform the design of future behavioral and structural level interventions in tobacco control. Our findings suggest that comprehensive approach involving health system and community intervention in tobacco control could have a high payoff. At the same time, our findings strongly suggest that instituting tobacco control interventions in a systematic way significantly increases the likelihood that health care providers will consistently intervene with patients who use tobacco to provide them with appropriate counselling and treatment services. 
